# Differences in Medial and Lateral Gastrocnemius Stiffness after Exercise-Induced Muscle Fatigue

**DOI:** 10.3390/ijerph192113891

**Published:** 2022-10-26

**Authors:** Prarthana Sanya Lall, Abdulrahman M. Alsubiheen, Mishal M. Aldaihan, Hanuel Lee

**Affiliations:** 1 Graduate School, Department of Physical Therapy, College of Health Science, Gachon University, Incheon 21936, Korea; 2 Department of Rehabilitation Sciences, College of Applied Medical Sciences, King Saud University, Riyadh 11451, Saudi Arabia; 3 Department of Physical Therapy, College of Health Science, Gachon University, Incheon 21936, Korea

**Keywords:** eccentric exercise, muscle fatigue, myotonometer, stiffness, shear wave elastography

## Abstract

Muscles are affected at the cellular level by exercised-induced fatigue, inducing changes in their stiffness. Examining muscle stiffness can improve the knowledge of various pathologic conditions, such as pain and injury. The objective of this study was to examine the stiffness of the medial gastrocnemius (MG) muscle and the lateral gastrocnemius (LG) muscle to determine the changes in stiffness, and to assess the differences in the stiffness between the MG and the LG, as affected by muscle fatigue measured using shear wave elastography (SWE) and a MyotonPRO after inducing muscle fatigue. A total of 35 healthy young adults participated in the study. The stiffness of the MG and the LG were assessed before and after a muscle fatigue protocol (MFP), which included three sets of 50 eccentric contractions of the calf muscles of the dominant leg, at rest, and at maximum voluntary contraction (MVC). The measurements were taken with SWE and the MyotonPRO simultaneously. Compared to baseline, the resting stiffness of the MG and the LG significantly increased immediately, 24 h, and 48 h after muscle fatigue (*p* < 0.05); however, during MVC, the stiffness of the MG decreased (*p* < 0.05) and that of the LG showed no change (*p* > 0.05). When the stiffness of the MG and the LG were compared before and after the MFP, changes in the stiffness of the MG were significantly greater than those in the LG (*p* < 0.05). This signifies that the MG was more affected by the exercise-induced muscle fatigue than was the LG. The assessment of musculoskeletal tissue and its characteristics, before and after eccentric exercise, is crucial in the prevention of overuse injuries associated with repeated exposure to both low and high levels of force.

## 1. Introduction

Skeletal muscles are the most vital source of human body movements [1]. To perceive the significance of muscle function, it is important to understand the mechanical properties of skeletal muscles [2].

The prime synergists are the triceps surae muscle for plantar flexion, having dissimilar architectural properties, such as differences in fascicle length, pennation angle, and muscle length. In comparison, the gastrocnemius muscle crosses both the knee and ankle, being a two-jointed muscle, whereas the soleus is a single-jointed muscle [3]. In most studies, it has been presumed that changes seen in the medial gastrocnemius (MG) would act as a representation of the whole triceps surae muscle; however, studies have shown differences between the MG and the lateral gastrocnemius (LG) in terms of muscle architecture after eccentric exercise. The reason for this could be due to the uneven sharing of the load between the muscles. In addition, eccentric exercise can cause more damage to short fibers than to long fibers, and considering the different fascicular geometry of the triceps surae (MG, LG, and soleus), each muscle can be affected by the exercise differently [4]. In a previous study by Shinohara et al., greater stiffness was seen in the MG than in the soleus at rest and in contraction states with the knee in extension, which suggests that the involvement of the bi-articular gastrocnemius muscle is greater with the knee in the extended position than in the flexed position [5].

During physical activities such as running and hopping, the MG and LG muscles show spring-like characteristics [6,7]. Tissue stiffness is represented as a mechanical property on the longitudinal axis during passive stretching, as measured by tissue resistance [1]. This is generally important for transmitting and absorbing motion energy in sports [2]. Kalkhoven et al. recently demonstrated in a study that, for football players, greater stiffness in the medial gastrocnemius muscle seems to be more beneficial in their athletic performance [8]. The propulsive force and stability of the ankle joint during movements occur through the integrated activation of the agonist and antagonist muscles [9]. The tibialis anterior muscle acts as an antagonist to the gastrocnemius, and electromyography (EMG) reveals that, during maximum voluntary contraction (MVC), the agonist and antagonist muscles are simultaneously activated [10]. The estimation of EMG and the force relation of the antagonist muscle can be used to detect a loss of strength in the agonist muscle [11].

The center of pressure (COP) is defined as “the center of all the external forces acting on the plantar surface of the foot” [12]. The biomechanical effort of the muscles around the ankle joint is to keep the COP inside the base of support (BOS), which is, in simple terms, the area of the body in contact with the supporting surface [13]. The COP, during dynamic movement, normally moves from lateral (from heel) to medial (great toe) for stability and the maintenance of balance [14]. This is attributable to the fact that the medial compartment of the knee and foot are susceptible to larger loads during movement which induces the MG to work more than the LG [15].

Eccentric muscle contractions are an important part of daily activities [3]. Unaccustomed eccentric contractions cause exercise-induced muscle damage, which typically presents as muscle soreness or fatigue [4,5] Exercise-induced muscle fatigue is a reversible loss of muscle contractility and can occur directly after exercise (acute muscle fatigue, which can be relieved quickly with rest or lifestyle changes) or over a long period of high-intensity exercise (delayed exercise-induced fatigue, which persists for months and is not relieved with rest). Recovery from fatigue is defined as the return of the function of the muscle to the baseline level after being fatigued. Fatigue is a functional state that occurs after prolonged exercise, especially exercise involving eccentric contractions [16,17,18].

In recent years, shear wave elastography (SWE) has evolved with favorable results in discovering and diagnosing pathological conditions of muscles [19,20]. The instrument acts as a quantitative technique for analyzing tissue stiffness [21], and it also acts as a direct measurement tool as shear waves arise in the tissue due to focused acoustic impulses with velocities equal to the square root of the shear elastic modulus of the tissue. It calculates the stiffness of tissue as Young’s modulus by analyzing the velocity of induced shear wave propagation from a region of interest (ROI) in a defined muscle or soft tissue [6,22,23,24].

Although SWE is a reliable tool in measuring tissue stiffness, it has some limitations. It requires technical expertise and higher maintenance costs, which make it less suitable for wider clinical use [6,19]. The MyotonPRO provides a much easier way to measure the stiffness of soft biological tissues, such as muscles, tendons, and ligaments [25]. It conveniently and rapidly assesses the mechanical properties by digitally palpating the superficial skeletal muscles and tendons [6,26]. The probe of the instrument records the oscillation of the muscle to calculate the stiffness [19]. Although investigations have shown promising results for the clinical use of the MyotonPRO, robust research on its measurement properties and clinical use is still lacking [19,27,28].

Therefore, the purpose of this study was to examine the effect of exercise-induced muscle fatigue and recovery in MG and LG stiffness using SWE and MyotonPRO and to visualize the differences in the levels of stiffness between the MG and the LG as affected by muscle fatigue.

## 2. Materials and Methods

### 2.1. Participants

A total of 35 healthy college students (20 males and 15 females), with a body mass index (BMI) between 18 kg/m^2^ and 30 kg/m^2^ [19], were voluntarily recruited through posters, announcements, and advertisements. Participants were included if they had no central or peripheral disorders [29], a low-to-moderate self-reported physical activity level [30], had not undergone any surgery in the prior 6 months, and had neither a recent lower-limb injury nor any use of analgesic medications [31]. Participants were excluded if they had any current injury or pain in their lower limbs, a history of corticosteroid injections, or any potential health risks associated with exercise, as reported in the physical activity readiness questionnaire (PAR-Q). None of the participants were athletes or had undergone regular resistance training or vigorous exercise. They were asked to avoid activities thought to affect the mechanical properties of muscles and tendons (such as jumping, stretching, weightlifting, and running) on the day before the measurements were taken [30,32]. Participants were asked to refrain from exercises, medications, massages, using ice packs, and using hot fermentation, and they were also asked to keep their physical activity to a minimum until the measurements had been completed. This study was approved by the Institutional Review Board of Gachon University (1044396-201909-HR-175-01) and performed in accordance with the guidelines of the Declaration of Helsinki. All participants were asked to provide written informed consent before participating in the study.

### 2.2. Experimental Procedure and Measurement Position

**Figure 1:**
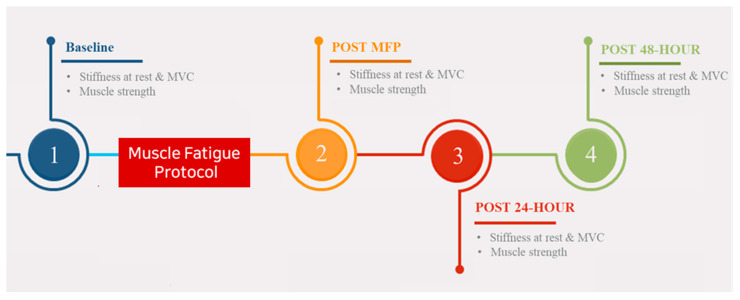
Experimental procedure. Abbreviations: MVC, maximum voluntary contraction; MFP, muscle fatigue protocol.

Upon arrival at the laboratory, the participants were asked to rest comfortably in a room with a normal temperature for 10 min to stabilize their body conditions. Before starting the experiment (baseline), their heights and weights were measured. The participants were instructed on how to contract their muscles, and they were asked to practice MVC for familiarization. All participants underwent a total of four measurements: at baseline, immediately after the MFP, 24 h after the MFP, and 48 h after the MFP (Figure 1). They were asked to lie prone with their feet off the table, and the area to be tested was exposed. The specific location for the stiffness measurement was determined and marked with a permanent marker. The skin over the muscles was exposed for the measurements of the stiffness. The levels of MG and LG muscle stiffness were assessed at a location four fingerbreadths below the popliteal crease [19]. To cross-check the MG and LG measurements, an ultrasound transducer was positioned transversely on the muscle belly and then moved from medially to laterally. The highest position of muscle thickness was observed in the image, and the appropriate the area was marked on the skin for stiffness measurements by both of the instruments. To measure muscle stiffness using the MyotonPRO and SWE, the participants were asked to relax the MG and LG for 30 s. Thereafter, they contracted the MG and LG as previously instructed, holding the contraction for 10 s each during the measurements. The participants were given 30 s between the two measurement methods to relax the muscles.

### 2.3. Muscle Fatigue Protocol

**Figure 2:**
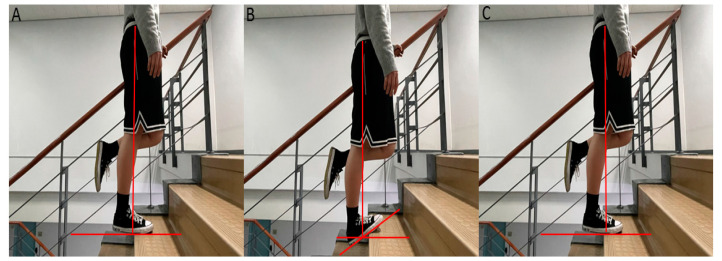
While standing on the dominant leg (**A**), the gastrocnemius muscle was eccentrically contracted by lowering the heel as much as possible with the knee extended (**B**), and then returned to baseline to repeat the contraction (**C**). The vertical line represents the line of gravity, and the horizontal lines represent the ankle at the neutral and maximum degrees of dorsiflexion.

The muscle fatigue protocol of the current study was adopted and modified from previous studies [29,33]. The protocol intended to induce muscle fatigue in the dominant leg, and it comprised three sets of 50 eccentric contractions of the calf muscles (a total of 150 contractions). Each set was separated by a gap of 2–3 min. The participants were made to stand on a step on their dominant leg and lower the heel, eccentrically contracting their calf muscles until the maximum degree of dorsiflexion was attained and the contraction was repeated. The non-dominant leg was flexed at the knee joint. To return to the baseline position, so as to stop the set, the participant used the other leg only to lift their body weight. During the eccentric contraction, the participant was asked to fully extend the knee of the dominant leg to maintain greater activation of the gastrocnemius muscle [34] (Figure 2). All the participants were asked to complete the contractions as much as was possible. A metronome set at 60 beats per minute was used along with verbal commands to assist and encourage the participants to complete the contractions in the required time. Each set was stopped when the participant was not able to further complete the contractions at the set pace.

### 2.4. Outcome Measures

#### 2.4.1. Shear Wave Elastography

An ACUSON S3000 ultrasound device (Siemens Healthcare, Erlsangen, Germany) and a 9 MHz linear probe were used for SWE measurements. During the acquisition of the data, the probe was placed parallel to the muscle fibers and was positioned with a generous amount of coupling gel and very light pressure for 8–10 s on the muscle bellies of the targeted muscles. SWE generates both a color map overlay that represents a qualitative elastogram, as well as an objective stiffness measurement. The transducer was kept stationary for 8–10 s on the marked area of the muscles for the acquisition of the stiffness measurement. A total of 5 ROIs in each image was assessed, and the average of the 5 ROIs was recorded for further analysis. The elastographic images were developed using the colors red and blue. Good intra-rater reliability for the stiffness measurements of the muscles of the lower limb was demonstrated by previous research [35].

#### 2.4.2. MyotonPRO

The mechanical measurements of tissue stiffness were examined using a MyotonPRO (Myoton AS, Tallin, Estonia). The probe was placed on the previously marked points of the MG and LG. The researcher placed the probe on the site of measurement, and the MyotonPRO completed the measurement. The impulse generated by the probe on the muscle lasts for 15 milliseconds, with a light mechanical force of 0.6 N [19,36]. The mechanical force given to the underlying tissue creates motion in the form of a damped free oscillatory decay that is measured by the device. The device screen shows the calculated mechanical stiffness measurement in (N/m). Reliability estimates for stiffness measures range between 0.898 and 0.986 in the gastrocnemius muscle [37].

#### 2.4.3. Hand-Held Dynamometer

The MicroFET2 handheld dynamometer (HHD) (Hogan Scientific, Salt Lake City, UT, USA) was used to assess the amount of force generated by the MG and LG before and after the MFP. It was also used during contraction of the MG and LG to standardize the force and to deliver biofeedback to the subjects. The HHD was firmly secured to a wall with adhesive Velcro strips to allow adjustments and stability. Handheld dynamometry has been shown to have good-to-excellent reliability in measuring isometric lower limb strength and power in healthy subjects [38].

### 2.5. Sample Size Estimation

G Power 3.1.9.7 software (Heinrich-Heine-University Dusseldorf, Düsseldorf, Germany) was used to determine the sample size. The alpha error probability and power were set at 0.05 and 0.90, respectively. The effect size was set at 0.25 based on Cohen’s effect size f, for a total of four levels of measurement [39]. As a result, a total sample size of 30 was estimated; however, an additional 5 participants were included to provide for unanticipated attrition.

### 2.6. Statistical Analysis

Statistical analyses were performed using Statistical Product and Service Solutions software for Windows 8.1 (version 23.0; SPSS, IBM Corp., Armonk, NY, USA). All data are presented as mean ± standard deviation (SD), and the Shapiro–Wilk test was used to test for normal distribution. The Friedman test was performed to assess changes in the stiffness of the MG and LG across all measurement time points at each muscle state (resting and MVC), as well as for strength. Wilcoxon signed-rank tests were used to assess the difference in stiffness between the MG and LG. The level of significance was set at α = 0.05.

## 3. Results

The general characteristics of the participants are presented in Table 1. All participants provided a negative response in the PAR-Q, indicating that there was no potential health risk associated with the MFP.

### 3.1. Changes in Muscle and Tendon Stiffness at Rest and during Contraction across All Measurement Time Points

The differences in the stiffness of the MG and LG across all measurement time points at rest and during contraction are summarized in Table 2. The stiffness measured by the MyotonPRO showed significant changes over time (*p* < 0.05) in the MG and LG at rest. In the contraction phase, the MG showed a significant change in stiffness (*p* < 0.05), whereas the LG showed no significant change across all the measurement time points (*p* > 0.05).

In the MG and LG, a significant change in the resting mean stiffness was observed over time (*p* < 0.05), respectively, as measured by SWE. The contraction stiffness in the MG was found to be statistically significant over time (*p* < 0.05), whereas the contraction stiffness of the LG showed no change (*p* > 0.05).

### 3.2. Differences between the Stiffness of Medial and Lateral Gastrocnemius

**Figure 3:**
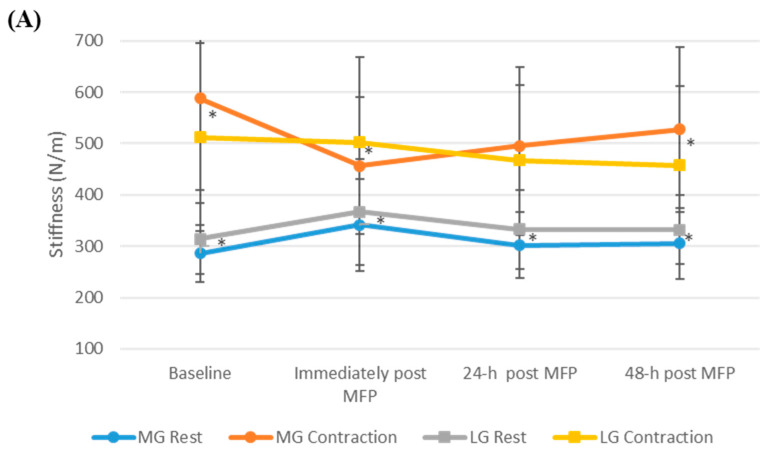

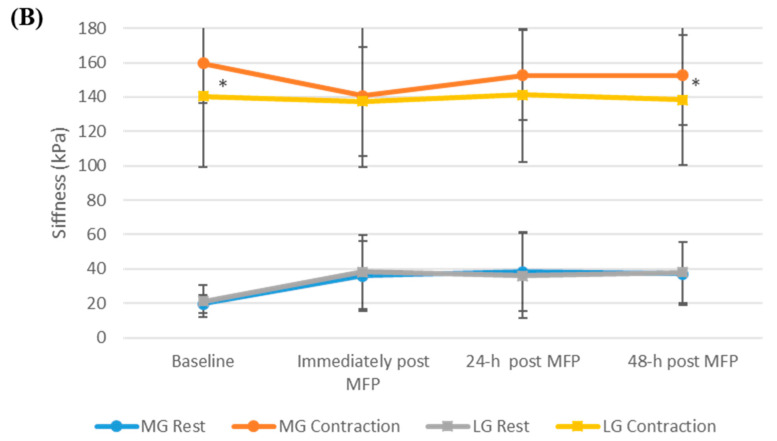
Differences between the stiffness of the medial and lateral gastrocnemius muscles, at rest and in contraction states, across all the measurement time points as measured using (**A**) MyotonPRO and (**B**) shear wave elastography. * Significant differences between the muscles at 0.05 level.

The differences are summarized in Table 3. At rest, there were significant differences between the MG and the LG at baseline, immediately post-MFP, 24 h post-MFP, and 48 h post-MFP (*p* < 0.05), when measured using the MyotonPRO, whereas with SWE, no significant difference was seen between the two muscles at any of the measurement time points (*p* > 0.05). With both the instruments, there was a significant difference between the contraction stiffness of the MG and the LG (*p* < 0.05), both at baseline and immediately after the MFP (*p* < 0.05), with the MyotonPRO, but not with SWE. At 24 h after the MFP, the muscles showed no significant differences (*p* > 0.05), but, at 48 h, a significant difference was seen between the MG and the LG (*p* < 0.05), as obtained by both of the instruments (Figure 3).

### 3.3. Changes in Strength across All Measurement Time Points

A significant change in strength was observed across all measurement time points (*p* < 0.05). A significant decrease was seen from baseline (243.87 ± 46.54) to immediately after the MFP (210.52 ± 52.52, *p* < 0.001) although no other changes were recorded at 24 h and 48 h after the MFP.

## 4. Discussion

The objective of the present study was to determine whether there are changes in the stiffness of the MG and the LG before and after exercised-induced muscle fatigue (immediately, 24 h, and 48 h after the MFP), as well as to determine which muscle was affected more. We hypothesized that, after the MFP, the stiffness of the gastrocnemius muscle will change, both at rest and during MVC, and the MG will be affected more than the LG. Our results showed a significant increase in the stiffness of the MG and LG in the resting state as well as immediately, 24 h, and 48 h after the MFP, as compared to baseline; however, the stiffness of the MG significantly decreased during MVC across all measurement time points, whereas that of the LG showed no change.

The baseline resting stiffness values of the MG and LG, as measured with both instruments in this study, were concordant with those reported in other studies [6,19,33]. In our study, the stiffness of the MG and LG, as measured with both methods, revealed a significant increase from baseline across all measurement time points in the resting state of the muscle. However, with the MyotonPRO, a significant difference was also observed in the MG and the LG at 24 h and 48 h after the MFP, as compared with measurements performed immediately after the MFP. No significant change was observed between 24 h and 48 h after the MFP. Lacourpaille et al. observed increased stiffness of the elbow extensors at long-muscle lengths at 1 h and 48 h after eccentric exercise; however, this increase was observed only when the muscles were stretched. No significant changes were found when the muscles were at rest, and the stiffness values were observed to return to the baseline values after 48 h [40]. Andonian et al. detected a significant decrease in the stiffness of the quadriceps muscle at rest (without differentiating between the heads of the muscles but mostly in the vastus lateralis) following an extreme mountain ultra-marathon and observed recovery 48 h later [41]. Similarly, Green et al. found that the stiffness of the MG increased after an eccentric exercise protocol, peaked at 48 h, and recovered within 1 week, when measured using magnetic resonance elastography [2]. However, in a study by Guilhem et al., the stiffness of the MG, measured using SWE, significantly increased from baseline after an eccentric exercise protocol (10 sets of 30 repetitions) although there was no significant difference at 48 h between the pre- and post-measures at rest [42]. An increase in muscle compliance due to disruption and progressive overextension of a few sarcomeres caused by repetitive eccentric contractions and decreased strength immediately after exercise probably indicates that metabolic muscle fatigue is not related to muscle damage [43,44]. This explains why a more significant change in stiffness was observed after exercise, whereas at 24 h and 48 h, stiffness was observed to decrease but remained below the baseline level (which denotes recovery from fatigue).

In this study, the MG showed significant changes in stiffness during MVC, whereas the LG showed no significant change. With SWE, a significant decrease was observed from baseline to immediately after the MFP, whereas stiffness measured with the MyotonPRO showed a significant decrease from baseline to immediately after the MFP and 24 h after the MFP. An increase in stiffness was observed from immediately after the MFP to 48 h after the MFP, indicating the recovery of the muscle. This decrease in stiffness after exercise can be explained by the loss of MVC after the MFP, signifying that the muscles were fatigued. Muscle fatigue can be defined as an exercise-induced decline in the maximal force capacity of the muscles involved [45]. When the muscles of the participants were fatigued, they were unable to fully contract the muscles, resulting in decreased stiffness values after the MFP.

Exercise-induced pain and the action of the agonist and antagonist muscles are additional aspects to consider. Eccentric contraction is known to induce pain, fatigue, swelling, tenderness, and stiffness in muscles although the most common immediate effect is pain [46]. Arendt-Nielsen and Graven-Nielsen reported that pain in agonist muscles caused a decrease in the electromyography (EMG) activity of agonist muscles and increased the EMG activity of antagonist muscles during dynamic contraction. This functional adaptation by the muscles can restrict movement, decrease the force of contraction, and protect the painful muscle by minimizing its muscular activity [47]. Our results showed a decrease in the stiffness of the MG and LG (agonist) across all measurement time points, as compared to baseline during MVC, as a result of muscle fatigue and pain, which, in turn, may have resulted in increased activity of the tibialis anterior (antagonist). This may have caused a decrease in the strength and efficiency of the muscle to fully contract and, accordingly, to a decrease in the measured stiffness values. These may be the reasons leading to a significant decrease in stiffness in the MG, but not in the LG, during MVC in our study.

In addition, a further analysis was executed so as to observe the difference in the stiffness before and after exercise-induced muscle fatigue between the MG and the LG. The resting stiffness as measured using the MyotonPRO demonstrated that the LG had significantly greater stiffness than the MG at baseline, immediately post-MFP, 24 h post-MFP, and 48 h post-MFP. SWE, on the other hand, showed no significant difference between the two muscles across all of the measurement time points. Although at rest, stiffness of the LG was greater than that of the MG, but a larger change in stiffness was seen in MG across all the levels of measurement, which explains why the MG was affected more by fatigue than the LG.

Several previous studies measured the stiffness between the MG and the LG using the MyotonPRO and reported greater stiffness of the LG, similar to the present study, but none of the studies observed differences between the muscles, nor did they focus on the reason for the greater stiffness of the LG at rest [6,15,26,48]. There are very few architectural differences between the muscles. First, the LG has a longer fiber length than the MG, which indicates that, in a series, the number of sarcomeres is the largest for the muscle, and the longer the muscle fiber is, the greater the velocity potential for that muscle is [3,49,50]. The MyotonPRO induces a damped natural oscillation in the muscle and records dynamic stiffness based on the maximum acceleration of the damped oscillation [51]. As acceleration is defined as the rate of change of velocity, the greater stiffness of the LG as measured with the MyotonPRO can be estimated, as velocity is a priority for the LG [49]. Secondly, the LG has a bipennate structure, whereas the MG has a unipennate structure [52], and greater stiffness has been observed in bipennate muscles [53,54]. For these reasons, our results show greater stiffness in the LG at rest.

Additionally, fiber orientation can affect the measurement of stiffness. The type of muscle fiber can be a potential factor that might affect stiffness, considering that type I fibers are stiffer than type II fibers. Nonetheless, this cannot explain the greater LG stiffness, as the percentage of the fiber type is the same in both of the muscles. In addition, the perimysium and endomysium can affect muscle stiffness at rest [3,55]. Hence, the greater stiffness of the LG at rest, as found in our study, can be explained.

At contraction, the stiffness of the MG was greater than that of the LG, but immediately after the MFP, the stiffness of the MG decreased significantly, which shows that the MG was fatigued. There can be various reasons that could denote that the MG was fatigued more during the MFP than was the LG in the present study. The physiological cross-sectional area of the MG is greater, as are the muscle volume and pennation angle, because of which, it generates more than 70% of the strength, twice as much as LG [3,26,49,52]. As stated, there is a positive relationship between the level of muscle contraction and the stiffness [56]; accordingly, our results show that the contraction stiffness of the MG was greater than the LG at baseline. Immediately after the MFP, the stiffness of the MG decreased, while the LG showed no change, denoting the extent to which the MG was affected by the exercise and was fatigued more than the LG. At 48 h, the stiffness of the MG was greater than that of the LG, showing gradual increase in stiffness, which, in turn, indicates recovery from exercise-induced muscle fatigue. Males and females illustrated a similar pattern of stiffness changes between the MG and the LG in our study.

During the MFP, only the ball of the foot was in contact with the ground, which formed the BOS throughout the exercise. The COP, during dynamic movement, normally moves from lateral (from heel) to medial (great toe) for stability and the maintenance of balance [14]. If the COP shifts toward the toes, the activation of the gastrocnemius muscle increases [57,58], precisely on the medial side, as the line of force during movement acts under the COP, passing from medial to the knee joint. The medial side of the foot and knee sustain a greater load than does the lateral side; thus, the sharing of the load between the synergistic muscles is not similar. Taking into account that the transfer of ground reaction force is crucial during dynamic movements, for that reason, the eccentric load on the MG was more than that of the LG [4,12,15,59]. Moreover, short-muscle fibers are more prone to damage caused by eccentric exercise than are long-muscle fibers [4], which further explains why the MG was affected more, as is seen in our results.

The limitations of this study must be addressed. First, subcutaneous fat was not assessed while taking measurements with the MyotonPRO, and muscles covered with >20 mm subcutaneous fat may affect stiffness measurements [6,51]. Second, the muscle force during the contraction was not quantitatively measured. Future studies should attempt to include EMG analysis to quantify or standardize stiffness changes after fatigue. Although we attempted to exclude the impact of the soleus muscle by keeping the knee at full extension during the sets of the MFPs, it still could have impacted the overall stiffness measurements. The study could not monitor the center of gravity and ground reaction forces during the MFP, and that could have been a factor in appropriately determining differences between males and females.

## 5. Conclusions

Muscle fatigue is a common phenomenon that affects both athletic performance and the performance of various other activities. In this study, the stiffness of the MG decreased, and that of the LG showed no change. Therefore, the measurement of stiffness can strengthen the understanding of muscle fatigue and the changes it causes to the muscle itself. The assessment of musculoskeletal tissue and its characteristics before and after eccentric exercises is crucial in the prevention of overuse injuries associated with repeated exposure to low or high levels of force. It can provide background information that is important for developing effective training strategies. Overall, the results of our study may have relevant applications for elite sports practice and for musculoskeletal rehabilitation.

## Figures and Tables

**Table 1:** General characteristics of the participants (*N* = 35).

Characteristics	Value (Mean ± SD)
Age (years)	23.91 ± 2.74
Sex (n), Female	15 (43 %)
Height (cm)	167.17 ± 9.02
Weight (kg)	63.58 ± 9.76
BMI (kg/m^2^)	22.65 ± 2.13

Abbreviations: SD, standard deviation; BMI, body mass index.

**Table 2:** Changes in muscle stiffness in the resting and contraction states across all measurement time points (*N* = 35).

		MyotonPRO (N/m) (Mean ± SD)				
		Baseline	Immediately Post-MFP	24 h Post-MFP	48 h Post-MFP	W
MG	R	285.83 ± 59.96	341.49 ± 90.11 *	301.46 ± 64.09 *^,$^	305.37 ± 68.88 *^,$^	0.50
	C	587.46 ± 177.89	456.40 ± 133.24 *	495.26 ± 154.27 *	527.74 ± 160.46 ^$^	0.22
LG	R	314.40 ± 69.20	366.77 ± 102.77 *	332.74 ± 77.35 *^,$^	331.60 ± 67.03 *^,$^	0.31
	C	512.03 ± 182.58	502.49 ± 164.94	467.51 ± 146.30	475.51 ± 155.18	0.03
		**SWE (kPa)** **(Mean ± SD)**				
		**Baseline**	**Immediately Post-MFP**	**24 h Post-MFP**	**48 h Post-MFP**	**W**
MG	R	19.68 ± 5.25	33.02 ± 12.75 *	38.34 ± 22.96 *	37.25 ± 18.51 *	0.36
	C	161.57 ± 19.69	136.59 ± 24.19 *	152.55 ± 26.11	152.65 ± 28.92	0.15
LG	R	21.21 ± 9.15	36.33 ± 18.50 *	36.06 ± 24.90 *	37.90 ± 17.60 *	0.33
	C	140.44 ± 41.20	139.62 ± 29.55	141.14 ± 39.01	138.21 ± 37.93	0.03

Abbreviations: SD, standard deviation; SWE, shear wave elastography; R, resting; C, contraction; MFP, muscle fatigue protocol; MG, medial gastrocnemius; LG, lateral gastrocnemius; W, Kendall’s W, effect size of Friedman test; r, effect size of Wilcoxon signed-rank test. * Significant changes in stiffness values from baseline at the 0.001 level. ^$^ Significant changes in stiffness from immediately after the MFP at the 0.001 level.

**Table 3:** Differences between muscles at rest and in contraction states across all measurement time points (*N* = 35).

MyotonPRO (N/m) (Mean ± SD)					
		Baseline	Immediately Post-MFP	24 h Post-MFP	48 h Post-MFP
MG	R	285.83 ± 59.96	341.49 ± 90.11	301.46 ± 64.09	305.37 ± 68.88
	C	587.46 ± 177.89	456.40 ± 133.24	495.26 ± 154.27	527.74 ± 160.46
LG	R	314.40 ± 69.20 *	366.77 ± 102.77 *	332.74 ± 77.35 *	331.60 ± 67.03 *
	C	512.03 ± 182.58 *	502.49 ± 164.94 *	467.51 ± 146.30	475.51 ± 155.18 *
**SWE (kPa)** **(Mean ± SD)**					
		**Baseline**	**Immediately Post-MFP**	**24 h Post-MFP**	**48 h Post-MFP**
MG	R	19.68 ± 5.25	33.02 ± 12.75	38.34 ± 22.96	37.25 ± 18.51
	C	161.57 ± 19.69	136.59 ± 24.19	152.55 ± 26.11	152.65 ± 28.92
LG	R	21.21 ± 9.15	36.33 ± 18.50	36.06 ± 24.90	37.90 ± 17.60
	C	140.44 ± 41.20 *	139.62 ± 29.55	141.14 ± 39.01	138.21 ± 37.93 *

Abbreviations: SD, standard deviation; SWE, shear wave elastography; R, resting; C, contraction; MFP, muscle fatigue protocol; MG, medial gastrocnemius; LG, lateral gastrocnemius; AT, Achilles tendon. * Significant changes between the MG and LG at the 0.05 level.

## Data Availability

The datasets generated during this study are available from the corresponding author upon reasonable request.
